# Effect of Protein-Rich Breakfast on Subsequent Energy Intake and Subjective Appetite in Children and Adolescents: Systematic Review and Meta–Analysis of Randomized Controlled Trials

**DOI:** 10.3390/nu13082840

**Published:** 2021-08-18

**Authors:** Meijuan Qiu, Yu Zhang, Zheng Long, Yuna He

**Affiliations:** 1 National Institute for Nutrition and Health, Chinese Center for Disease Control and Prevention, No. 29 Nanwei Road, Xicheng District, Beijing 100050, China; qiumj_smile@163.com; 2 Department of Clinical Nutrition and Department of Health Medicine, Peking Union Medical College Hospital, Chinese Academy of Medical Sciences (CAMS) and Peking Union Medical College (PUMC), No. 1 Shuaifuyuan, Dongcheng District, Beijing 100730, China; zhangyu_a0849@163.com; 3 National Center for Chronic and Noncommunicable Disease Control and Prevention, Chinese Center for Disease Control and Prevention, No. 27 Nanwei Road, Xicheng District, Beijing 100050, China; jfh920068313@163.com

**Keywords:** protein, breakfast, subsequent energy intake, appetite, fullness, hunger, meta–analysis, children, adolescents

## Abstract

Breakfast has been labeled “the most important meal of the day”, especially for children and adolescents. Dietary protein intake may benefit and regulate appetite and energy balance. However, few meta–analyses have been conducted to examine the effect of protein–rich (PR) breakfast on both children and adolescents. This meta–analytic study was conducted to examine the effect of consuming a PR breakfast on short–term energy intake and appetite in children and adolescents. PubMed, Embase, Cochrane Central Register of Controlled Trials, China Biology Medicine disc (CBM), and China National Knowledge Infrastructure (CNKI) were searched for randomized controlled trials (RCTs) published in January 1990–January 2021. The inclusion criteria applied were RCTs in children and adolescents (7–19 year) comparing PR breakfast consumption with normal protein (NP)/traditional breakfast consumption. Finally, ten studies were included in the analysis, eight studies examined the effect of consuming PR breakfast on SEI (*n* = 824), and nine studies examined the effect on appetite (fullness = 736, hunger = 710). Our meta-analysis using the random–effects model shows that participants assigned to consume PR breakfast had lower SEI (MD, −111.2 kcal; 95% CI: −145.4, −76.9), higher fullness (MD, 7.4 mm; 95% CI: 6.0, 8.8), and lower hunger (MD, −8.5 mm; 95% CI: −9. 7, −7.3) than those assigned to consume NP/traditional breakfast. However, there was considerable inconsistency across the trial results. Our review suggests that the consumption of PR breakfast could be an excellent strategy for weight management by declining SEI and suppressing appetite, and provides new evidence of the relationship between energy balance and obesity. However, since most eligible studies were of low quality, the results ought to be interpreted cautiously.

## 1. Introduction

The prevalence of obesity has risen continuously over the past decades in low– and middle–income countries as well as in many high-income countries [1,2,3]. Obesity is a worldwide health concern in children and adolescents resulting from long-term imbalance of energy (energy intake > expenditure intake) [4]. Obesity and obesity–related disorders such as cardiovascular diseases and type–2 diabetes is increasing steadily worldwide [5,6,7]. Moreover, the main risk factors for attributable DALYs globally, in 2019, was child and maternal malnutrition, which accounted for 11.6 % of all global DALYs that year [8].

Strategies for obesity prevention and management are multiple, including bariatric surgery, drug therapies, physical activity, and so on [9]. Among them, dietary recommendation is an effective strategy for the prevention and treatment of obesity among children and adolescents [10]. Particularly, breakfast, the most important meal of the day, has played a pivotal role in weight management and energy balance [11]. Furthermore, dietary protein is essential to the health of individuals of all ages, and is especially critical for the growth and development of children and adolescents. Thus, protein–rich (PR) breakfast consumption might be a useful strategy for weight management [12]. However, there is no consensus on the definition for PR breakfast. Given this lack of consensus, and to maximize identified articles for this review, we defined a PR breakfast as any breakfast containing more protein than the normal protein (NP)/traditional breakfast, and there were no restrictions on protein sources, protein doses, protein type, and macronutrient composition of breakfast.

PR breakfast promotes weight loss in children and adolescents possibly through regulating appetite and subsequent food intake (SFI) [13,14,15,16,17], whereas the effect of PR breakfast on appetite and subsequent energy intake (SEI) is inconsistent. Recent studies among children and adolescents have challenged the conclusion of PR breakfast and by checking the findings of six randomized controlled trials (RCTs) that did not show the effect on reductions of SEI [13,14] and subjective appetite [14,15,16,17]. To our knowledge, RCTs’ meta-analysis has not been conducted to evaluate the effects of PR breakfast on appetite and SEI in both children and adolescents.

Thus, this study aimed to search for the evidence of children and adolescents from RCTs to identify the effect of PR breakfast on subjective appetite and SEI for a better understanding of the relationship between energy balance and obesity, focused on the studies published in the last thirty years.

## 2. Materials and Methods

Our systematic review followed the PRISMA (Preferred Reporting Items for Systematic Reviews and Meta–Analyses) statement [18].

### 2.1. Search Strategy and Study Selection

We gathered literature from January 1990 through January 2021 by conducting a systematic search in PubMed, Embase, Cochrane Central Register of Controlled Trials, China Biology Medicine disc (CBM), and China National Knowledge Infrastructure (CNKI). We also searched ClinicalTrials.gov in order to identify any unpublished or ongoing RCTs. We adjusted a 31–year search limit because dietary patterns from three decades ago may have changed dramatically over the past several decades [19]. Additionally, relevant reviews and studies of all references were also screened for other relevant citations. We restricted the search to RCTs of children and adolescent studies. The search strategy is described in detail in Table 1 and Table 2. Two reviewers examined inclusion and exclusion criteria independently by screening the titles, abstracts, and then the full–text of the articles. Search terms included “Breakfast”, “Child, Preschool”, “Minors”, “Students”, “randomized controlled trial”, “ready to eat cereals/RTEC”, etc.

### 2.2. Selection Criteria

The studies included in this review meet the following criteria: (1) subjects include children and adolescents aged 7–19 years old with no restrictions regarding sex, races, or health status; (2) having the intervention that after overnight fasting the subjects consumed a single breakfast meal; (3) studies with explicit breakfast composition, specifically protein content; (4) investigating the effect of PR breakfast on SEI or subjective appetite components (fullness or hunger); (5) use visual analogue scale questionnaire (VAS) to evaluate different aspects of subjective appetite; (6) reporting means and standard error (SE) or standard deviation (SD) or 95% confidence intervals (CI) for SEI and/or fullness and hunger; (7) randomized controlled or crossover trials study design; and (8) studies published in English or Chinese. The articles were excluded if they meet any of the exclusion criteria: (1) articles without sufficient data like reviews, guidelines, case reports, non–human studies, etc.; (2) participants with diabetes, cancer, or other specific conditions that impacted subjective appetite or postprandial metabolism; (3) trials among groups that used other interventions such as health education and promotion, exercise, drug treatments, and dietary supplements; (4) articles without sufficient relevant outcome data; and (5) full–text articles or originals were not available.

### 2.3. Data Extraction

Two reviewers (Qiu and Zhang) independently extracted data of the included studies and any disagreements were resolved by discussion until resolved, including: (1) First authors’ names, publication year, country, study design, duration; (2) Sex and age of participants, body mass index (BMI) percentage of female participants, subject health status; (3) Intervention and control group (Composition of the whole breakfast); (4) Subsequent lunch intake details; and (5) Study results for SEI and subjective appetite (including fullness or hunger). A third reviewer (Long) checked the extracted data. All reported SE were converted to SD. If data were not available in digital form, we used WebPlotDigitizer (WebPlotDigitizer. 2020. https://automeris.io/WebPlotDigitizer/; accessed date: 30 January 2021) to approximatively estimate it from corresponding graphs. WebPlotDigitizer is an open-source, semi–automatic digitization, web–based, free online tool. All the available images files from the original publications were imported to WebPlotDigitizer. The study results for SEI and subjective appetite (including fullness or hunger) were then extracted.

### 2.4. Appraisal of the Quality of Studies

Two reviewers (Qiu and Zhang) independently evaluated the quality of eligible studies using the Cochrane Collaboration’s tool (ROB 1). This tool assesses the risk of bias according to the following domains: randomization, allocation concealment, blinding of participants and personnel, blinding of outcome assessment, missing outcome data, selective reporting, and other sources of bias. The risk of bias for each item was classified as high, low, or unclear. A trial with low risk of all items was rated the overall quality at low risk of bias, at least one item was at high risk was judged as having a high risk of bias overall, otherwise the overall quality was at unclear risk.

### 2.5. Data Synthesis

Mean differences ± SDs of SEI and subjective appetite, comparing consuming PR breakfast with NP/traditional breakfast were used to calculate the overall effects of eligible studies. Differences in SEI and appetite were analyzed using weighted mean difference (WMD). Due to clinical and methodological between–study heterogeneity, all effect size calculations used a random–effects model. Between–study heterogeneity was evaluated using I^2^. Subgroup analysis was based on sex (girl, boy, and both), study design (cross–over and parallel), subject health status (non–overweight and overweight), economic status of country (High–income country and Medium– and Low–income country). Publication bias of SEI was assessed by funnel plots. Sensitivity analysis was performed by the leave–one–out method on studies that may cause bias in the results. All statistical analyses were conducted in R 4.0.3 (packages meta and robvis). *p* < 0.05 was considered statistically significant.

## 3. Results

### 3.1. Literature Search and Screening

**Figure 1:**
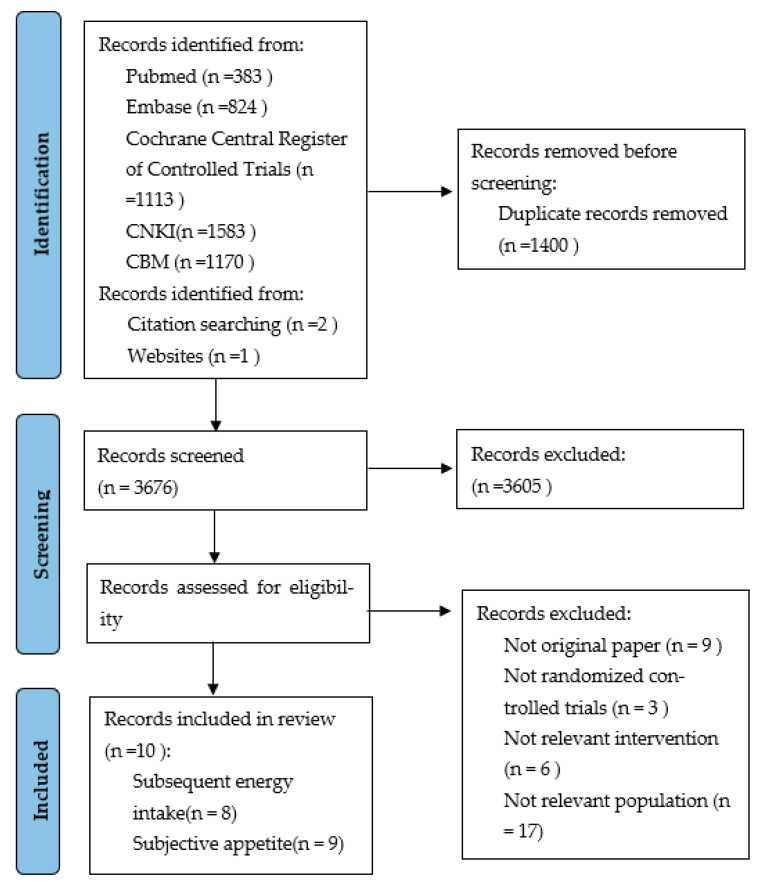
Flow diagram of the literature search process.

The search of the five electronic databases identified 5076 records of which 1403 articles were remained after duplicate removal. After screening titles and abstracts, 3605 studies were excluded because they did not meet inclusion and exclusion criteria. Then, 71 studies underwent full–text screening, and 61 studies were excluded after full-text evaluation. Finally, ten studies were included in the analysis (Figure 1) [13,14,15,16,17,20,21,22,23,24]. We searched two ongoing trials from ClinicalTrials.gov that potentially meet our inclusion criteria and are included in future updates of this review (NCT01192100 and NCT03146442).

### 3.2. Study Design Characteristics

The characteristics of the included studies are summarized in Table 3. The interventions of all included studies are a PR breakfast. The included studies were published between 2010 and 2020. The sample size ranged from 13 to 156 subjects, with a mean age ranged from 9 to 19 years. Most studies were carried out in the high–income countries [13,14,15,16,20,21,22] and the middle–income countries [17,23,24]. All studies were conducted on healthy children and adolescents. Six trials included specifically with obesity/overweight subjects [13,17,21,22,23,24]; the remaining trials included a population with any weight range, including normal weight, overweight, and obese subjects [13,14,15,16,20]. Of the 10 studies, eight studies examined the effect on SEI [13,14,15,16,17,20,23,24] and nine studies examined it on subjective appetite measured by VAS [13,14,15,16,17,21,22,23,24].

### 3.3. Risk of Bias across Studies

**Figure 2:**
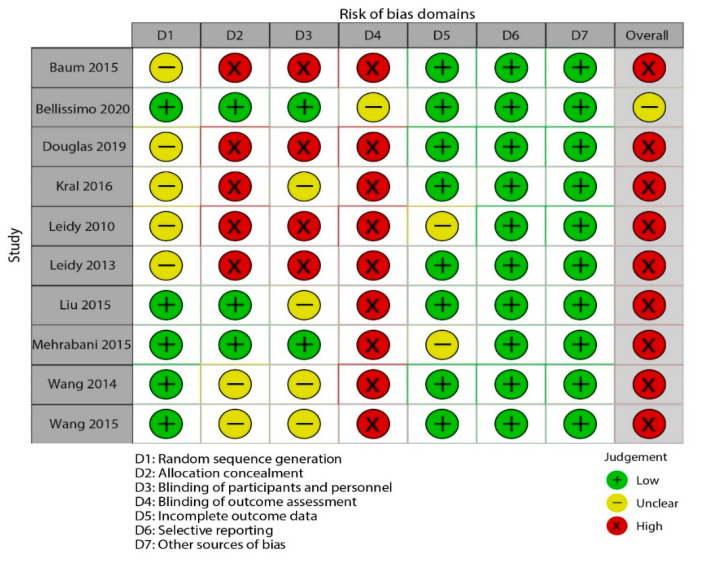
Study quality and risk of bias assessment of included studies in the meta−analysis (traffic light).

**Figure 3:**
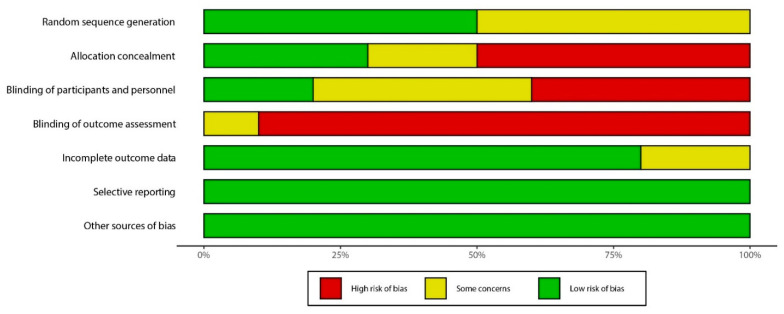
Study quality and risk of bias assessment of included studies in the meta−analysis (summary).

The risk of bias assessments for all included studies was presented in Table 4, Figure 2 and Figure 3. Due to lack of allocation concealment, blinding of participants and personnel, and blinding of subjective and objective outcome assessment, the primary issues were at a high risk of bias among the ten RCTs. Most RCTs reported information regarding randomization sequence generation was at unclear risk. Of ten included studies, nine were categorized as high risk, and one as unclear risk.

### 3.4. Findings from Meta–Analysis

#### 3.4.1. Protein–Rich Breakfast and Subsequent Energy Intake

**Figure 4:**
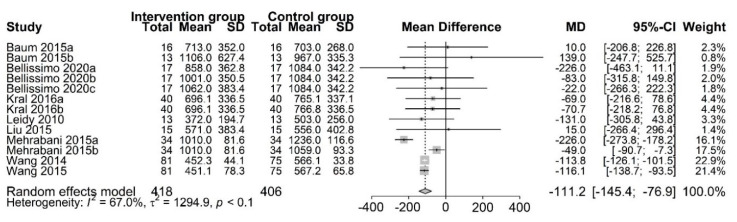
Random–effects meta–analysis of relationships between protein–rich breakfast and subsequent energy intake (kcal). Data for Baum 2015a [13] are based on non–overweight participants, whereas data for Baum 2015b [13] are based on overweight participants. Bellissimo 2020a [20], Bellissimo 2020b [20], and Bellissimo 2020c [20] are based on different subsets of subjects who identified as different dose protein breakfast consumers. Kral 2016a (Oatmeal vs. Scrambled eggs) [15], Kral 2016b (Cereal vs. Scrambled eggs) [15], Mehrabani 2015a (LFM vs. W) [23], and Mehrabani 2015b (LFM vs. AJ) [23] are based on different subsets of subjects who identified as different control groups, respectively. Other studies were defined as Leidy 2010 [16], Liu2015 [14], Wang2014 [17], and Wang 2015 [24], respectively.

**Figure A1:**
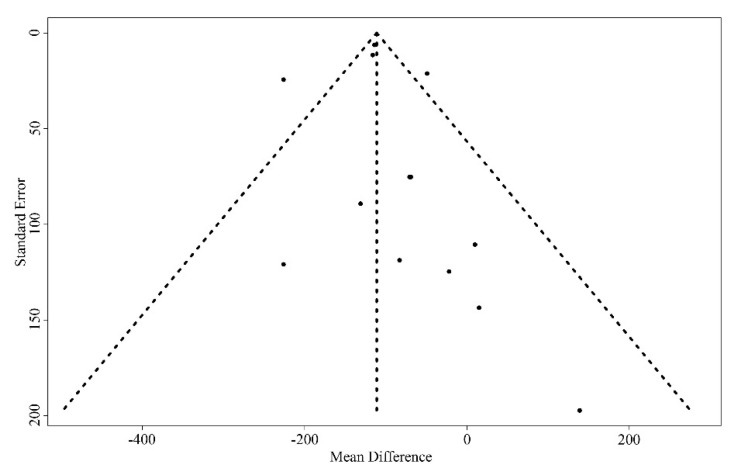
Funnel plot for random effects meta-analysis of mean difference in subsequent energy intake (kcal).

The effect of PR breakfast on SEI was examined in eight studies [13,14,15,16,17,20,23,24]. At the end of the trials (range 9 days to 3 months), we observed that participants who were assigned to consume PR breakfast had a lower SEI than those assigned to consume NP/traditional breakfast (MD, −111.2 kcal; 95% CI: −145.4 to −76.9; *p* < 0.01) (Figure 4), namely, consuming PR breakfast elicits the decrease of SEI. However, we did detect considerable inconsistency across trial results (Tau^2^ = 1294.9, I^2^ = 67.0%, Q = 36.3). The funnel plot showed some asymmetry (Figure A1). After the elimination of one trial, the results were largely robust to the traditional sensitivity analysis. The heterogeneity was significantly reduced (MD, −100.0 kcal; 95% CI: −120.5 to −79.5; Tau^2^ = 213.6, I^2^ = 24.0%, Q = 14.5). In addition, we performed a subgroup analysis based on study design, sex, economic status of country, and baseline body mass index (Table 5). Thus, we presumed that the trial of Mehrabani et al. [23] was the source of heterogeneity.

#### 3.4.2. Breakfast and Subjective Appetite

##### Protein–Rich Breakfast and Fullness

**Figure 5:**
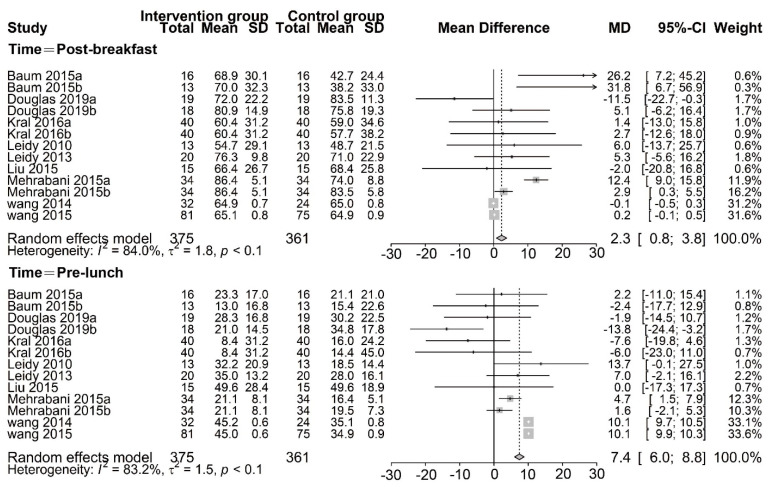
Random–effects meta–analysis of relationships between protein–rich breakfast and fullness (mm). Data for Baum 2015a are based on non–overweight participants, whereas data for Baum 2015b are based on overweight participants. Bellissimo 2020a [20], Bellissimo 2020b [20], and Bellissimo 2020c [20] are based on different subsets of subjects who identified as different dose protein breakfast consumers. Data for Douglas 2019a [21] are based on different subsets of subjects who habitually skipped breakfast, whereas data for Douglas 2019b [21] are based on different subsets of subjects who habitually consumed breakfast. Kral2016a (Oatmeal vs. Scrambled eggs) [15], Kral2016b (Cereal vs. Scrambled eggs) [15], Mehrabani 2015a (LFM vs. W) [23], and Mehrabani 2015b (LFM vs. AJ) [23] are based on different subsets of subjects who identified as different control groups, respectively. Other studies were defined as Leidy 2010 [16], Liu2015[14], Wang2014 [17], and Wang 2015 [24], respectively.

Fullness was reported according to the effect of PR breakfast in nine studies, including two time points of post–breakfast and pre–lunch [13,14,15,16,17,21,22,23,24].We found that participants who were assigned to consume PR breakfast had a higher fullness than those assigned to consume NP/traditional breakfast, in random–effects meta–analysis of the post–breakfast (MD, 2.3 mm; 95% CI:0.8, 3.8; *p* < 0.01) and pre–lunch group (MD, 7.4 mm; 95% CI: 6.0, 8.8; *p* < 0.01) (Figure 5), although there was substantial inconsistency across trial results (Tau^2^ = 1.8, I^2^ = 84.0%, Q = 74.7 and Tau^2^ = 1.5, I^2^ = 83.2%, Q = 71.4, respectively). The meta–analysis results for the pooled effects of the post–breakfast and pre–lunch groups were robust in the sensitivity analysis. Similarly, we also conducted a subgroup analysis (Table 6). To assess the impact of study design, we exclude the crossover design of the trial and found a large change in the mean difference (post–breakfast: 5.8 mm, 95% CI: 3.9, 7.7; pre–lunch: 2.39 mm, 95% CI: 0.3, 4.5). However, we found that it did not have a significant impact on the heterogeneity of the post-breakfast and pre–lunch group.

##### Protein–Rich Breakfast and Hunger

**Figure 6:**
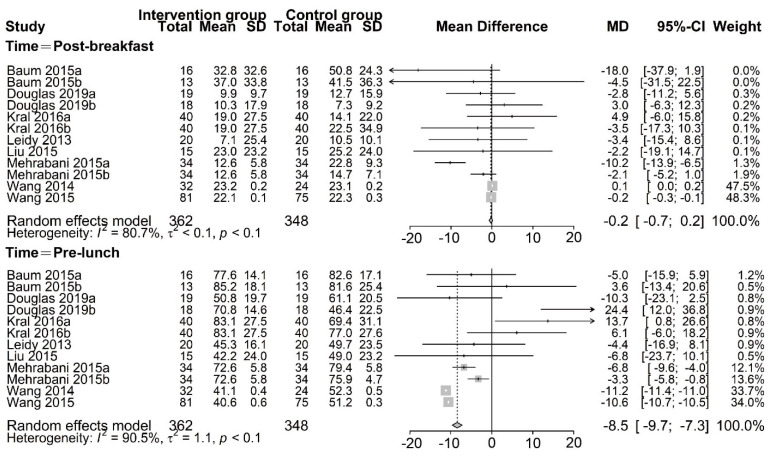
Random–effects meta–analysis of relationships between protein–rich breakfast and hunger (mm). Data for Baum 2015a [13] are based on non–overweight participants, whereas data for Baum 2015b [13] are based on overweight participants. Bellissimo 2020a [20], Bellissimo 2020b [20], and Bellissimo 2020c [20] are based on different subsets of subjects who identified as different dose protein breakfast consumers. Data for Douglas 2019a [21] are based on different subsets of subjects who habitually skipped breakfast, whereas data for Douglas 2019b [21] are based on different subsets of subjects who habitually consumed breakfast. Kral 2016a (Oatmeal vs. Scrambled eggs) [15], Kral 2016b (Cereal vs. Scrambled eggs) [15], Mehrabani 2015a (LFM vs. W) [23], and Mehrabani 2015b (LFM vs. AJ) [23] are based on different subsets of subjects who identified as different control groups, respectively. Other studies were defined as Liu2015 [14], Wang2014 [17], and Wang 2015 [24], respectively.

The effect of PR breakfast on hunger was examined in eight studies [13,14,15,17,21,22,23,24]. A random–effects meta–analysis revealed that the hunger did not differ between trials in the post–breakfast group (MD, −0.2 mm; 95% CI: −0.7, 0.2; *p* < 0.01) (Figure 6). However, we found that participants who were assigned to consume PR breakfast had a lower hunger than those assigned to consume NP/traditional breakfast in the pre–lunch group (MD, −8.48 mm; 95% CI: −9.7, −7.3; *p* < 0.01) (Figure 6), although there was significant inconsistency across trial results (Tau^2^ = 1.1, I^2^ = 90.5%, Q = 116.0). The meta–analysis result of the pre–lunch group was steady in the sensitivity analysis. Likewise, the results of all other subgroup analyses were not significant (Table 7).

## 4. Discussion

This systematic review and meta–analysis of 10 studies examined the effect on SEI and appetite in children and adolescents consuming a PR breakfast. We found new evidences to support the opinion that the PR breakfast consumption decreased SEI compared with consuming NP/traditional breakfast. Furthermore, there was an evidence indicated that consumption of a PR breakfast can increase fullness and decrease hunger. When we conducted a subgroup analysis based on study design, sex, economic status of country, and baseline body mass index, the results of the pre–lunch group were similar. In addition, this review and meta–analysis provided the first evidence demonstrating the effect of PR breakfast on SEI and subjective appetite components (hunger and fullness), and provided new evidences of the relationship between energy balance and obesity.

### 4.1. Principal Findings

Energy imbalance seems to be an independent risk factor in the etiology of obesity [25]. Meta–analysis of RCTs showed decreased SEI in participants who consumed a PR breakfast compared with those who consumed NP/traditional breakfast among children and adolescents. The sensitivity analysis indicated that the trial of Mehrabani et al. [23] was responsible for the most of the heterogeneity. We compared Mehrabani 2015a with Mehrabani 2015b and found that the breakfast composition and energy contribution were greatly different between the trials. In addition, the included studies [15,16,17,19,22,23] showed that consuming a PR breakfast reduced SEI, but not total energy intake (TEI) [15,16,20]. However, the previous study supported a negative association between dietary protein and TEI [26]. Thus, the significant reduction in SEI in participants suggests that serving a PR breakfast may be a strategy to regulate energy balance in children and adolescents.

We also found that fullness was higher and hunger was lower in groups consuming PR breakfast than those consuming an NP/traditional breakfast among children and adolescents. And we observed that the effect of a PR vs. an NP/traditional breakfast had a higher fullness at post–breakfast. Some included studies [13,14,17,24] had reported appetite at 30 min post–breakfast. Of these, only one trial [13] reported that the normal weight children consuming PRO breakfast had significantly lower glucose values at 30 min than those children consuming CHO breakfast, this suggested that diets higher in protein and lower in carbohydrate had been shown to improve glycemic control. Thus, appetite regulation is likely one of the mechanisms that are responsible for better glycemic control. The subgroup analysis was performed in our research to investigate the possible explanations for the heterogeneity of satiety in the post–breakfast group and the pre–lunch group. The results of fullness may be affected by some aspects of the study design. However, study design does not influence the results of hunger. This difference may be due to the difference in time intervals. One of the included studies, conducted in Iran, found the greatest differences in appetite scores at 4 h after breakfast intake and these differences remained significant at 5 h [23]. The finding may be explained via food between the preloads and their subsequent meals, while the related indicators were not measured in other studies [13,14,15,16,17,20,21,22,23]. In addition, a recent systematic review indicated that consuming a high–protein diet may influence subjective appetite by enhancing fullness, while hunger-reduction observed in the high protein diet did not convert to appetite [27].

We saw methodological differences across the trials of the length of measurement period, energy content, and macronutrient composition of breakfast. And these differences may in part account for the heterogeneities. The effects of PR breakfast on SEI and appetite are greatly influenced by various protein doses and forms [28]. There is no consensus on breakfast protein recommendations for children and adolescents. Most included studies were conducted with adolescents, and the recommended dietary allowance for dietary protein is 0.85 g protein kg^−1^ day^−1^ for adolescents aged 14–18 years [29]. However, in our included studies protein dose of interventions ranged from 12.2 g to 58 g. In addition, protein type may also be a critical factor impacting the heterogeneities. In the most trials, the protein was administered in a semi–solid/solid form. Four of the included studies examined the effect of an egg breakfast [14,15,17,24]. Previous studies showed complete proteins can drive thermogenesis, thus affecting the synthesized effect [30]. Another explanation might be due to the differences in breakfast size, meal frequency and habitual breakfast patterns [31,32]. Taken together, the data did not support enough the potential mechanisms of the effect of dietary protein breakfast. Further high–quality studies are needed to fill the important gap.

According to the protein leverage hypothesis, the body preferentially consumes protein in three main nutrients (carbohydrate, fat, and protein). If a breakfast lacks adequate protein, then we had to attempt to acquire a higher amount of protein from more food, leading to an increased risk of obesity [33]. Furthermore, protein increases diet–induced thermogenesis (DIT) more than carbohydrates and fats in adults due to the high energy costs related to protein synthesis and changes in substrate utilization conducing to fat oxidation [34,35]. Two of the included studies examined carbohydrate oxidation and fat oxidation [13,19]. One of the two studies, conducted in non–overweight individuals, found the protein–rich treatment had lower carbohydrate oxidation and greater fat oxidation compared to the control breakfast. However, the other study conducted in non–overweight and overweight subjects, respectively, researchers found greater fat oxidation but no difference in carbohydrate oxidation between different meals. Furthermore, the differences in results between the trials are also likely to be attributed to confounders from other compositions in breakfast, such as fiber and fat [36]. However, previous studies showed that protein has better appetite suppressive effects than other nutrients [37]. Another study indicated that dietary protein content was negatively related to TEI irrespective of whether fat or carbohydrate was the diluents of protein [25]. Thus, it is presumed that the consumption of higher protein at breakfast could assist in weight management because of declined SEI and suppressed appetite.

### 4.2. Quality of Evidence

For some reasons, we consider the quality of evidence to be low. All included studies were at unclear risk, or high risk of bias in at least one risk of bias item. More strictly conducted trials could draw more decisive conclusions. We also observed considerable heterogeneity among the results of subjective components (hunger and fullness). Firstly, although the VAS can serve as a useful supplementary method to measure food intake, it is lack of uniform scale and appetite rating is subjective [38]. Furthermore, the heterogeneity may be partly due to the age difference of intervention objects. The degree of refinement of brain structure and function varies in children and adolescents of different ages, although we narrowed the inclusion of age [39].

Most of the studies included in this review were conducted in the US, UK, and Canada [13,14,15,16,19,20,21]. Protein sources and nationally habitual breakfast patterns in these countries may differ greatly from those in other countries that do not follow the western dietary patterns, such as China or Japan. Veldhorst et al. [40] found that different protein sources can affect SEI and subjective appetite. Thus, the findings concerning SEI and appetite should be interpreted with caution.

### 4.3. Limitations

This meta–analysis had some limitations. At first, a further obvious limitation is that there is not adequate numbers of literatures and subjects included in the review, which will be resolved in the future. Second, the search strategy should have reported short–term energy intake or appetite as search terms. This omission might influence the number of included articles. Additionally, we set a 31–year limit according to the previous studies [19,41]. However, we should ideally have performed search from inception until January 2021 and then do meta–regression for year of reporting to ascertain such change, which will provide a time series like interpretation. Third, the included trials lasted from 9 days up to 3 months. Although the divergence in SEI between PR breakfast eaters and NP/traditional breakfast eaters was about 111 kcal. In some studies [15,16,21], total energy intake (TEI) was not different between the groups, whereas in others [13,14,20,21,22,23,24] TEI was not measured. And short– and long–term protein consumption could also produce different effects on appetite [42]. Thus, it is hard to draw conclusions about SEI and subjective appetite based on existing results. More long–term trials are needed to identify whether these changes cause long–term alterations in routine energy regulation and appetite control when the PR breakfast is consumed in daily. Fourth, the included studies examined a series of hormones associated with energy balance and appetite regulation, including ghrelin and PYY (serum peptide YY). Six of the included studies examined changes in hormones [14,16,17,21,22,24]. The levels of ghrelin and PYY did not differ significantly between the intervention and control groups. We have no explanation for this phenomenon, and this needs to be further researched.

## 5. Conclusions

As the quality of the eligible studies was mostly low, the results ought to be interpreted cautiously. Currently, the meta–analysis reveals consuming a protein–rich breakfast has an impact on decreased subsequent energy intake, decreased hunger and increased fullness among children and adolescents. And our review provides a better understanding of the relationship between energy balance and obesity by regulation of short–term energy intake or appetite. More high–quality RCTs are needed to prove whether those children and adolescents against obesity should consume protein–rich breakfast and identify the suitable dosage of protein.

## Figures and Tables

**Table 1:** Description of the PICOS (Participants, Interventions, Control, Outcomes) statement.

PICOS	Descriptions
Participants	Children and adolescents older than 7 and younger than 19 years; Both sexes; All nationalities
Interventions	The intervention group consumed a protein-rich breakfast; No restrictions regarding the dose or intervention duration were applied.
Control/ Comparator group	The control group consumed a normal protein or traditional breakfast;
Outcomes	Subsequent energy intake or subjective appetite components (fullness and hunger)
Setting	Randomized controlled or crossover trials

**Table 2:** Search strategy for Pubmed.

1	((((“Breakfast”[Mesh]) OR (((((((((((((Breakfasts[Title/Abstract]) OR (Breakfast Time[Title/Abstract])) OR (Breakfast Times[Title/Abstract])) OR (Time, Breakfast[Title/Abstract])) OR (Times, Breakfast[Title/Abstract])) OR (Morning Meal[Title/Abstract])) OR (Meals, Morning[Title/Abstract])) OR (Morning Meals[Title/Abstract])) OR (meal timing[Title/Abstract])) OR (Cereal[Title/Abstract])) OR (RTEC[Title/Abstract])) OR (Ready To Eat Cereals[Title/Abstract])) OR (breakfast cereal[Title/Abstract]))) AND ((“Breakfast”[Mesh]) OR (((((((((((((Breakfasts[Title/Abstract]) OR (Breakfast Time[Title/Abstract])) OR (Breakfast Times[Title/Abstract])) OR (Time, Breakfast[Title/Abstract])) OR (Times, Breakfast[Title/Abstract])) OR (Morning Meal[Title/Abstract])) OR (Meals, Morning[Title/Abstract])) OR (Morning Meals[Title/Abstract])) OR (meal timing[Title/Abstract])) OR (Cereal[Title/Abstract])) OR (RTEC[Title/Abstract])) OR (Ready To Eat Cereals[Title/Abstract])) OR (breakfast cereal[Title/Abstract]))))
2	(((((“Child, Preschool”[Mesh]) OR (“Adolescent”[Mesh])) OR (“Minors”[Mesh])) OR (“Students”[Mesh])) OR (((((((((((((((((((((((((((Preschool Child[Title/Abstract]) OR (Children, Preschool[Title/Abstract])) OR (Preschool Children[Title/Abstract])) OR (Children[Title/Abstract])) OR (Adolescence[Title/Abstract])) OR (Teens[Title/Abstract])) OR (Teens[Title/Abstract])) OR (Teenagers[Title/Abstract])) OR (Teenager[Title/Abstract])) OR (Youth[Title/Abstract])) OR (Youths[Title/Abstract])) OR (Adolescents, Female[Title/Abstract])) OR (Adolescent, Female[Title/Abstract])) OR (Female Adolescent[Title/Abstract])) OR (Adolescents, Male[Title/Abstract])) OR (Female Adolescents[Title/Abstract])) OR (Adolescent, Male[Title/Abstract])) OR (Male Adolescent[Title/Abstract])) OR (Male Adolescents[Title/Abstract])) OR (juvenile adult[Title/Abstract])) OR (Minor[Title/Abstract])) OR (Minors[Title/Abstract])) OR (Student[Title/Abstract])) OR (School Enrollment[Title/Abstract])) OR (Enrollment, School[Title/Abstract])) OR (Enrollments, School[Title/Abstract])) OR (School Enrollments[Title/Abstract]))))
3	((randomized controlled trial [pt] OR controlled clinical trial [pt] OR randomized [tiab] OR placebo [tiab] OR clinical trials as topic [mesh: noexp] OR randomly [tiab] OR trial [ti]))
4	1 AND 2 AND 3
5	Filters: from 1 January 1990–1 January 2021

**Table 3:** Characteristics of included studies in the systematic review.

Author (Country, Year)	Study Design (Duration)	Participants			Intervention Group (Composition of the Whole Breakfast)	Control Group (Composition of the Whole Breakfast)	Composition of the Intervention Breakfast	Subsequent Lunch Intake	Appetite (Mean ± SD/SE *)	
		Population ^1^	Age (Mean ± SD)	BMI (Mean ± SD)					Intervention (mm)	Control (mm)
Baum (US, 2015) [13]	Crossover (9 days)	[*n* = 16; 44%], Nonoverweight	9.9 ± 1.2	16.7 ± 1.6	PRO (344 kcal, 18 g protein, 45 g CHO, 16 g sugars, 1 g fiber, 10.5 g fat)	CHO ^2^ (327 kcal, 3 g protein, 55 g CHO, 39 g sugars, 0.5 g fiber, 11 g fat)	Egg whites, butter, orange juice, white bread	Buffet-style meal served at 240 min	Hunger Post–breakfast: 32.8 ± 8.2 * Pre–lunch: 77.6 ± 3.5 * Fullness Post–breakfast: 68.9 ± 7.5 * Pre–lunch: 23.3 ± 4.2 *	Hunger Post–breakfast: 50.8 ± 6.1 * Pre–lunch: 82.6 ± 4.3 * Fullness Post–breakfast: 42.7 ± 6.1 * Pre–lunch: 21.1 ± 5.3 *
		[*n* = 13; 46%], Overweight/obese	9.5 ± 1.4	22.7 ± 4.0	PRO (344 kcal, 18 g protein, 45 g CHO, 16 g sugars, 1 g fiber, 10.5 g fat)	CHO * (327 kcal, 3 g protein, 55 g CHO, 39 g sugars, 0.5 g fiber, 11 g fat)	Egg whites, butter, orange juice, white bread	Buffet-style meal served at 240 min	Hunger Post–breakfast: 37.0 ± 9.4 * Pre–lunch: 85.2 ± 5.0 * Fullness Post–breakfast: 70.0 ± 9.0 * Pre–lunch: 13.0 ± 4.7 *	Hunger Post–breakfast: 41.5 ± 10.1 * Pre–lunch: 81.6 ± 7.0 * Fullness Post–breakfast: 38.2 ± 9.2 * Pre–lunch: 15.4 ± 6.3 *
Bellissimo (Canada, 2020) [20]	Crossover (25 days)	[*n* = 17; 47%], Nonoverweight	12.0 ± 1.65	20.8 ± 3.7	HP (450 kcal, 45 g protein, 30 g CHO, 2 g fiber, 17 g fat)MP (450 kcal, 30 g protein, 45 g CHO, 3 g fiber, 17 g fat)LP (450 kcal, 15 g protein, 61 g CHO, 5 g fiber, 17 g fat)	C (450 kcal, 7 g protein, 69 g CHO, 3 g fiber, 17 g fat)	Egg yolk, egg whites, butter, cheese, home fries, ketchup	Pizza lunch according to one’s preference served at 210 min	NA	NA
Douglas (US, 2019) [21]	Crossover (15 days)	[*n* = 19, 100%], Overweight	19 ± 1	29.0 ± 3.8	SKIP-HP (350 kcal, 35 g protein)	SKIP-NP (350 kcal, 13 g protein)	Yogurt parfaits, bagels, breakfast burritos, cereals, etc.	NA	Hunger Post–breakfast: 9.9 ± 9.7 Pre–lunch: 50.8 ± 19.7 Fullness Post–breakfast: 72.0 ± 22.2 Pre–lunch: 28.3 ± 16.8	Hunger Post–breakfast: 12.7 ± 15.9 Pre–lunch: 61.1 ± 20.5 Fullness Post–breakfast: 83. 5 ± 11.3 Pre–lunch: 30.2 ± 22.5
		[*n* = 18, 100%], Overweight	19 ± 1	28.9 ± 2.9	CONSUME-HP (350 kcal, 35 g protein)	CONSUME-NP (350 kcal, 13 g protein)	Yogurt parfaits, bagels, breakfast burritos, cereals, etc.	NA	Hunger Post–breakfast: 10.3 ± 17.9 Pre–lunch: 70.8 ± 14.6 Fullness Post–breakfast: 80.9 ± 14.9 Pre-lunch: 21.0 ± 14.5	Hunger Post–breakfast: 7.3 ± 9.2 Pre–lunch: 46.4 ± 22.5 Fullness Post–breakfast: 75.8 ± 19.3 Pre–lunch: 34.8 ± 17.8
Kral (US, 2016) [15]	Crossover (3 weeks)	[*n* = 40, 47.5%], Overweight/Nonoverweight	9.4 ± 0.8	NA Overweight or obese (45%)	Egg (350 kcal, protein % energy: 21)	Oatmeal (350 kcal, protein % energy: 14%)Cereal (350 kcal, protein % energy: 8%)	Scrambled eggs (prepared with 1/8 tsp. table salt), toasted whole wheat bread, diced peaches, and milk (1% fat)	Lunch (chicken nuggets, macaroni and cheese, green beans (prepared with 3 g of salted butter), ketchup, applesauce, chocolate chip cookies, and milk) served at 180 min	Hunger Post–breakfast: 19.0 ± 4.4 * Pre–lunch: 83.1 ± 4.4 * Fullness Post–breakfast: 60.4 ± 4.9 * Pre–lunch: 8.4 ± 4.9 *	Hunger (Oarmal) Post–breakfast: 14.1 ± 3.5 * Pre–lunch: 69.4 ± 4.9 * Fullness (Oarmal) Post–breakfast: 59.0 ± 5.5 * Pre–lunch: 16.0 ± 3.8 * Hunger (Cereal) Post–breakfast: 22.5 ± 5.5 * Pre–lunch: 77.0 ± 4.6 * Fullness (Cereal) Post–breakfast: 57. 7 ± 6.0 * Pre–lunch: 14.4 ± 7.1 *
Leidy (UK, 2010) [16]	Crossover (17 days)	[*n* = 13, 46%], Overweight/Nonoverweight	14.3 ± 1.1	23.5 ± 3.6	PR * (512 ± 26 kcal, 49.1 ± 2.5 g protein, 62.8 ± 3.2 g CHO, 30.7 ± 1.6 g sugar, 2.1 ± 0.1 g fiber, 7.5 ± 0.4 g fat)	PN * (513 ± 26 kcal, 18.1 ± 0.9 g protein, 95.3 ± 4.9 g CHO, 31.1 ± 1.6 g sugar, 2.0 ± 0.1 g fiber, 7.5 ± 0.4 g fat)	Whey Pancakes (whey protein powder, skim milk, margarine, egg–whites, butter, etc.)	Buffet lunch served at 240 min	Fullness Post–breakfast: 54.7 ± 8.1 * Pre–lunch: 32.1 ± 5.8 *	Fullness Post–breakfast: 48.7 ± 6.0 * Pre–lunch: 18.5 ± 4.0 *
Leidy (US, 2013) [22]	Crossover (5 weeks)	[*n* = 20, 100%], Overweight	19 ± 4.5	28.6 ± 3.1	HP (350 kcal, 35.1 g protein, 35.1 g CHO, 18 g sugar, 6.1 g fiber, 7.8 g fat)	NP (350 kcal, 13 g protein, 57 g CHO, 18 g sugar, 6.1 g fiber, 7.8 g fat)	Egg, Beef, Dairy, Plant–based, etc.	NA	Hunger Post–breakfast: 7.1 ± 5. 7 * Pre–lunch: 45.3 ± 3.6 * Fullness Post–breakfast: 76.3 ± 2.2 * Pre–lunch: 35.0 ± 2.9 *	Hunger Post–breakfast: 10.5 ± 2.3 * Pre–lunch: 49.7 ± 5.3 * Fullness Post–breakfast: 71.0 ± 5.1 * Pre–lunch: 28.0 ± 3.6 *
Liu (US, 2015) [14]	Parallel (9 days)	[*n* = 15, 60%], Overweight/Nonoverweight	15.6 ± 4.26	NA Overweight or obese (40%)	Egg (342 kcal, 16.8 g protein, 32.2 g CHO, 16.6 g fat)	Bagel (336 kcal, 11 g protein, 48.6 g CHO, 10 g fat)	Scrambled, toast, jelly	Lunch (baked chicken, macaroni and cheese, green beans, mandarin oranges, rolls, and milk) served at 180 min	Hunger Post–breakfast: 23.0 ± 6.0 * Pre–lunch: 42.2 ± 6.2 * Fullness Post–breakfast: 66.4 ± 6. 9 * Pre–lunch: 49.6 ± 7.3 *	Hunger Post–breakfast: 25.2 ± 6.2 * Pre–lunch: 49.0 ± 6.0 * Fullness Post–breakfast: 68.44 ± 6. 7 * Pre–lunch: 49.55 ± 4. 9 *
Mehrabani (Iran, 2015) [23]	Crossover (16 days)	[*n* = 34, 0%], Overweight	11.14 ± 0.8	27.62 ± 2.7	LFM (401.24 kcal, 19.08 g protein, 49.055 g CHO, 0.458 g fiber, 15.407 g fat)	W (297.74 kcal, 10.931 g protein, 37.185 g CHO, 0.458 g fiber, 12.779 g fat) AJ (411.44 kcal, 11.276 g protein, 65.195 g CHO, 1.016 g fiber, 13.022 g fat)	Low–fat milk, Iranian whole wheat bread, Walnut, Low–fat cheese	Buffet-style meal served at 300 min	Hunger Post–breakfast: 12.6 ± 1.0 * Pre–lunch: 72.6 ± 1.0 * Fullness Post–breakfast: 86.4 ± 0.9 * Pre–lunch: 21.1 ± 1.4 *	Hunger (W) Post–breakfast: 22.8 ± 1.6 * Pre–lunch: 79.4 ± 1.0 * Fullness (W) Post–breakfast: 74.0 ± 1.5 * Pre–lunch: 16.4 ± 0.9 * Hunger (AJ) Post–breakfast: 14.7 ± 1.2 * Pre–lunch: 75.9 ± 0.8 * Fullness (AJ) Post–breakfast: 83.5 ± 1.0 * Pre–lunch: 19.5 ± 1.3 *
Wang (China, 2014) [17]	Parallel (9 days)	[*n* = 56, 46%], Overweight	14.1 ± 2.1	32.2 ± 1.7	Egg (386 kcal, 12.2 g protein, 29.3 g CHO, 15.9 g fat)	Steamed bread (386 kcal, 8.2 g protein, 44.7 g CHO, 11.5 g fat)	Boiled eggs, White rice, Milk	Lunch (pork with Chinese cabbage, apple, and rice, etc.) served at 240 min	Hunger Post–breakfast: 23.2 ± 0.2 Pre–lunch: 41.1 ± 0.4 Fullness Post–breakfast: 64, 9 ± 0.7 Pre–lunch: 45.2 ± 0.6	Hunger Post–breakfast: 23.1 ± 0.2 Pre–lunch: 52.3 ± 0.5 Fullness Post–breakfast: 65.0 ± 0.8 Pre–lunch: 35.1 ± 0.8
Wang (China, 2015) [24]	Parallel (3 months)	[*n* = 156, 49%], Overweight	14.3 ± 2.2	32.0 ± 1.7	Egg (386 kcal, 12.2 g protein, 29.3 g CHO, 15.9 g fat)	Steamed bread (386 kcal, 8.2 g protein, 44.7 g CHO, 11.5 g fat)	Boiled eggs, White rice, Milk	Lunch (pork with Chinese cabbage, apple, and rice, etc.) served at 240 min	Hunger Post–breakfast: 22.1 ± 0.1 Pre–lunch: 40.6 ± 0.6 Fullness: Post–breakfast: 65.1 ± 0.8 Pre–lunch: 45.0 ± 0.6	Hunger Post–breakfast: 22.30 ± 0.3 Pre–lunch: 51.20 ± 0.3 Fullness: Post–breakfast: 64.9 ± 0.9 Pre–lunch: 34.9 ± 0.9

^1^ [total number completed: % girl], subject health status; PRO: protein-based breakfast; CHO ^2^: carbohydrate-based breakfast; HP: high protein; MP: medium protein; LP: low protein; C: control; SKIP-HP: habitually skipped higher-protein breakfast; SKIP-NP: habitually skipped normal-protein breakfast; CONSUME-HP: habitually consumed higher-protein breakfast; CONSUME-NP: habitually consumed normal-protein breakfast; PR: protein-rich breakfast; PN: normal-protein breakfast; HP: high-protein breakfast; NP: normal-protein breakfast; LFM: a fixed breakfast with low-fat milk; W: a fixed breakfast with water; AJ: a fixed breakfast with apple juice; NA: Not Applicable; BMI: body mass index; CHO: Carbohydrate; US = United States; UK = United Kingdom; 1 kcal = 4.18 kJ; SD = standard deviation; SE * = standard error.

**Table 4:** Study quality and risk of bias assessment of included studies in the meta-analysis.

Study ID	Random Sequence Generation	Allocation Concealment	Blinding of Participants and Personnel	Blinding of Outcome Assessors	Incomplete Outcome Data	Selective Reporting	Other Bias	Overall Quality
Baum 2015	Unclear risk	High risk	High risk	High risk	Low risk	Low risk	Low risk	High risk
Bellissimo2020	Low risk	Low risk	Low risk	Unclear risk	Low risk	Low risk	Low risk	Unclear risk
Douglas2019	Unclear risk	High risk	High risk	High risk	Low risk	Low risk	Low risk	High risk
Kral2016	Unclear risk	High risk	Unclear risk	High risk	Low risk	Low risk	Low risk	High risk
Leidy2010	Unclear risk	High risk	High risk	High risk	Unclear risk	Low risk	Low risk	High risk
Leidy2013	Unclear risk	High risk	High risk	High risk	Low risk	Low risk	Low risk	High risk
Liu2015	Low risk	Low risk	Unclear risk	High risk	Low risk	Low risk	Low risk	High risk
Mehrabani2015	Low risk	Low risk	Low risk	High risk	Unclear risk	Low risk	Low risk	High risk
Wang2014	Low risk	Unclear risk	Unclear risk	High risk	Low risk	Low risk	Low risk	High risk
Wang2015	Low risk	Unclear risk	Unclear risk	High risk	Low risk	Low risk	Low risk	High risk

**Table 5:** Results of subgroup–analysis for subsequent energy intake (kcal) and protein–rich breakfast.

		Number of Comparisons	WMD (95% CI)	Heterogeneity I^2^ (%)	*p* between
Study–design					
	Crossover	10	−116.9 (−145.6, −88.3)	75%	*p* < 0.0001
	Parallel	3	−114.1 (−124.9, −103.4)	0%	0.66
Sex					
	Girl	0			
	Boy	2	−125.3 (−156.8, −93.9)	97%	*p* < 0.0001
	Both	11	−113.2 (−123.9, −102.6)	0%	0.82
Economic status of country					
High–income country		9	−70.09 (−137.8, −2.4)	0%	0.83
Medium–and–low–income country		4	−115.49 (−125.7, −105.3)	90%	*p* < 0.0001
Baseline body mass index (BMI)					
Non–overweight/Overweight		8	−76.70 (−145.5, −8.0)	0%	0.87
	Overweight/Obese	5	−115.31 (−125.5, −105.1)	88%	*p* < 0.0001

**Table 6:** Results of subgroup–analysis for fullness (mm) and protein–rich breakfast.

		Number of Comparisons	WMD (95% CI)	Heterogeneity I^2^ (%)	*p* between
Subgroup analyses for fullness and protein–rich breakfast (post–breakfast)					
Study–design					
	Crossover	10	6.0 (4.1, 7.9)	76%	*p* < 0.0001
	Parallel	3	0.1 (−0.1, 0.3)	0%	0.46
Sex					
	Girl	3	−0.3 (−6.7, 6.2)	65%	0.06
	Boy	2	6.4 (4.3, 8.5)	95%	*p* < 0.0001
	Both	7	−0.1 (−0.5, 0.3)	58%	0.03
Economic status of country					
High–income country		9	2.5 (−2.2, 7.1)	57%	0.02
Medium–and low– income country		4	0.2 (−0.0, 0.4)	95%	*p* < 0.0001
Baseline body mass index (BMI)					
Non–overweight/Overweight		5	5.9 (−1.8, 13.5)	29%	0.23
	Overweight/Obese	8	0.2 (−0.0, 0.4)	90%	*p* < 0.0001
Subgroup analyses for fullness and protein–rich breakfast (pre–lunch)					
Study–design					
	Crossover	10	2.4 (0.3, 4.5)	53%	0.02
	Parallel	3	10.1 (9. 9, 10.3)	0%	0.52
Sex					
	Girl	3	−1.8 (−7.9, 4.2)	76%	0.01
	Boy	2	3.4 (1.0, 5.8)	37%	0.21
	Both	8	10.1 (9.9, 10.3)	59%	0.02
Economic status of country					
High–income country		9	−0.8 (−5.1, 3.4)	46%	0.06
Medium–and low– income country		4	10.1 (9.9, 10.3)	90%	*P* < 0.0001
Baseline body mass index (BMI)					
Non–overweight/Overweight		5	0.6 (−5.9, 6.9)	33%	0.2
	Overweight/Obese	8	10.0 (9.8, 10.2)	88%	*P* < 0.0001

**Table 7:** Results of subgroup–analysis for hunger (mm) an d protein–rich breakfast (pre-lunch).

		Number of Comparisons	WMD (95% CI)	Heterogeneity I^2^ (%)	*p* between
Study–design					
	Crossover	9	−3.8 (−5.5, −2.0)	78%	*p* < 0.0001
	Parallel	3	−10.8 (−10.9, −10.6)	88%	0.0002
Sex					
	Girl	3	3.5 (−3.7, 10.8)	88%	0.0002
	Boy	2	−4.9 (−6.8, −3.0)	70%	0.07
	Both	7	−10.8 (−10.9, −10.6)	86%	*p* < 0.0001
Economic status of country					
High–income country		8	2.9 (−1.7, 7.5)	70%	0.0002
Medium–and low– income country		4	−10.7 (−10.9, −10.6)	95%	*p* < 0.0001
Baseline body mass index (BMI)					
Non–overweight/Overweight		4	2.3 (−4.0, 8.7)	52%	0.1
	Overweight/Obese	8	−10.7 (−10.9, −10.6)	92%	*p* < 0.0001

## Data Availability

All data (reviewed articles) used, were from already published empirical articles, retrieved from databases. All data generated or analysed during this study were included in this published article.
